# Moisturizing body milk as a reservoir of *Burkholderia cepacia*: outbreak of nosocomial infection in a multidisciplinary intensive care unit

**DOI:** 10.1186/cc6778

**Published:** 2008-01-31

**Authors:** Francisco Álvarez-Lerma, Elena Maull, Roser Terradas, Concepción Segura, Irene Planells, Pere Coll, Hernando Knobel, Antonia Vázquez

**Affiliations:** 1 Services of Intensive Care Medicine, Evaluation and Clinical Epidemiology, and Internal Medicine and Infectious Diseases, Hospital Universitari del Mar, Universitat Autònoma de Barcelona, Passeig Marítim 25-29, E-08003 Barcelona, Spain; 2 Service of Infectious Pathology, Laboratori de Referència de Catalunya, C/Selva 10, edifice INBLAU A, Parc de Negocis Mas Blau, E-08820 El Prat de Llobregat, Barcelona, Spain; 3 Service of Clinical Microbiology, Hospital Vall d'Hebron, Universitat Autònoma de Barcelona, Passeig Vall d'Hebron 119-129, E-08035 Barcelona, Spain; 4 Service of Clinical Microbiology, Hospital de la Santa Creu i Sant Pau, Universitat Autònoma de Barcelona, C/Sant Antoni Maria Claret 167, E-08025 Barcelona, Spain

## Abstract

**Background:**

An outbreak of severe nosocomial *Burkholderia cepacia *infections in patients admitted to intensive care unit (ICU), including investigation of the reservoir, is described.

**Methods:**

Over a period of 18 days, isolates of *Burkholderia cepacia *were recovered from different biological samples from five patients who were admitted to a multidisciplinary 18-bed intensive care unit. Isolation of *B. cepacia *was associated with bacteraemia in three cases, lower respiratory tract infection in one and urinary tract infection in one. Contact isolation measures were instituted; new samples from the index patients and adjacent patients were collected; and samples of antiseptics, eau de Cologne and moisturizing body milk available in treatment carts at that time were collected and cultured.

**Results:**

*B. cepacia *was isolated from three samples of the moisturizing body milk that had been applied to the patients. Three new hermetically closed units, from three different batches, were sent for culture; two of these were positive as well. All strains recovered from environmental and biological samples were identified as belonging to the same clone by pulsed-field gel electrophoresis. The cream was withdrawn from all hospitalization units and no new cases of *B. cepacia *infection developed.

**Conclusion:**

Moisturizing body milk is a potential source of infection. In severely ill patients, the presence of bacteria in cosmetic products, even within accepted limits, may lead to severe life-threatening infections.

## Introduction

*Burkholderia cepacia *is a nonfermenting Gram-negative aerobic bacillus that was until recently considered an opportunistic pathogen in oncological patients or in those with cystic fibrosis. This pathogen is associated with low morbidity and mortality despite high intrinsic resistance to numerous antimicrobial and antiseptic agents [1]. It is characterized by a capacity to survive in a large variety of hospital microenvironments, resulting in its dissemination via contaminated respiratory equipment, disinfectants, blood analyzers and running water supply [2-5]. In intensive care units (ICUs) outbreaks of *B. cepacia *in association with contaminated nebulizers [6], indigo-carmine dye in patients with nasogastric tubes [7], or mouth washings [8] have been reported.

Simultaneous detection of several isolations of this pathogen in the same service heralds the occurrence of an epidemic outbreak associated with a reservoir. Under such these circumstances it is advisable that an epidemiological study be conducted to identify the origin of the infection and the epidemiological chain. Here we describe an outbreak of episodes of severe infection caused by *B. cepacia *in a multidisciplinary Spanish ICU in which contaminated moisturizing body milk served as the reservoir and origin of the infection. Elimination of the reservoir was associated with eradication of *B. cepacia *from the hospital.

## Materials and methods

### Description of the ICU

Our institution is a 450-bed tertiary care teaching hospital in the city of Barcelona, Spain. The multidisciplinary ICU includes 18 beds in a semicircular distribution, with independent rooms that may be isolated by transparent glass doors. Rooms are equipped with individual sinks and dispensers of alcohol solution for cleansing of the hands without water. Six of the rooms have an independent air extraction system. The nursing staff includes one nurse for each two beds in all shifts and one certified nurse assistant for each five beds in all shifts. All personnel have received basic training for the invasive procedures that they perform, and written protocols for each procedure are available. Overall, patients are admitted to the ICU because of medical complications (45%) and ischaemic heart disease (35%), with a lower percentage of elective surgical patients (10%) and polytrauma patients (10%). In 2006, the mean (± standard deviation) Acute Physiology and Chronic Health Evaluation II score was 10.6 ± 6.5, and the mean length of ICU stay was 7.9 ± 8.3 days. Patients were mechanically ventilated for 47% of ICU days and had a urinary catheter for 75% of days.

The ICU participates annually in a national surveillance programme for nosocomial infections. In the year 2006, the rate of nosocomial infections related to invasive devices was 16.6 per 1,000 days of ICU stay (50th percentile for the national study, which was 15.1 per 1,000 days of ICU stay). In previous years no case of infection with *B. cepacia *in the ICU has been registered. Also, as part of the hospital surveillance programme for multiresistant pathogens, weekly surveillance cultures from patients at risk for multiresistant pathogens (ICU stay >7 days, use of broad-spectrum antibiotics, and use or two or more invasive devices) are carried out; during the 24 months preceding the outbreak, *B. cepacia *had not been identified in these samples.

### Description of the outbreak

The index cases were those patients in whom *B. cepacia *was isolated in one or more biological samples. *B. cepacia *isolates were classified as colonization or infection. The US Centers for Disease Control and Prevention definitions for nosocomial infections [9] were used. 'Outbreak' was defined as the simultaneous presence of four patients admitted to the ICU with positive cultures for *B. cepacia *(a further patient was later identified). The outbreak was detected through routine infection control surveillance.

In all cases, *B. cepacia *strains were isolated from clinical samples in standard culture media. Identification was performed using the biochemical tests MicroScan^® ^(Dade-Behring, West Sacramento, CA, USA) and API System (BioMerieux, Marcy l'Etoile, France). Microdilution (panel NC38, MicroScan^®^) and disk diffusion techniques were used for antibiotic susceptibility testing. New samples from the index patients and adjacent patients at greater risk for cross-transmission, including oropharyngeal mucosa, urine and bronchial aspirate samples, were collected. Samples of the antiseptic (iodine solution, 70% isopropyl alcohol and chlorhexidine), eau de Cologne and moisturizing body milk available in the treatment carts at that time were also collected and sent to the Laboratory of the Service of Microbiology (Unit of Food and Environmental Microbiology) of the Hospital Vall d'Hebron in Barcelona. These samples were cultured using the following media: blood agar, MacConkey agar, brain heart infusion agar, brain heart infusion agar supplemented with Tween 80, and liquid and solid media for anaerobic micro-organisms. The Vitek 2 system (BioMerieux) was used in the identification of the different pathogens. Strains isolated from environmental samples were frozen and, together with strains recovered from biological samples, were sent to the laboratory of microbiology of Hospital Santa Creu i Sant Pau, in Barcelona, for subsequent molecular typing by immunoelectrophoretic methods. Pulsed field gel electrophoresis (PFGE) of chromosomal DNA digested with *Spe*l was performed using Chef DRIII System apparatus (Bio-Rad, Richmond, CA, USA), under conditions appropriate for the enzyme. Lambda ladder PFGE marker (New England Biolabs, Beverley, MA, USA) was used as the standard marker. Analysis of PFGE profiles was conducted using the software Bio Image Whole Band Analyzer (Genomic Solutions, Ann Arbor, MI, USA).

The Committee of Infections of the hospital was notified of the occurrence of the outbreak. Informed consent from patients was not required because investigation of the outbreak, isolation measures and detection of the source of infection did not involve interventions other than those routinely performed in the care of patients under these circumstances.

In accordance with official recommendations of the government of Catalonia [10] and following the protocol implemented in the hospital, contact isolation measures were instituted. These included assigning patients to their own room, handwashing on entry and exit (with soap and water, and alcohol disinfection), use of disposable gowns and gloves, use of clinical materials exclusively for the patient (stethoscope and pulse oximeter) and visiting restrictions. Cleaning measures in the rooms were intensified, including use of single-use material or materials exclusive to each patient. Patients with local signs of infection and/or inflammatory systemic response were given one or more antibiotics, depending on results of antibiotic susceptibility testing. Every effort was made to increase universal precautions to avoid cross-transmission of micro-organisms, especially hand washing and use of alcohol solutions.

## Results

During a period of 18 days in August 2006, five patients admitted to a multidisciplinary ICU were identified in whom one or more strains of *B. cepacia*, with identical pattern of antibiotic susceptibility (sensitivity to ciprofloxacin, meropenem, piperacillin-tazobactam and co-trimoxazole; resistance to aminoglycosides, cephalosporin, imipenem, penicillins and aztreonam), were recovered from different biological samples. The individual details for each patient, including date of admission to the hospital, date of admission to the ICU and recovery of the first sample in which *B. cepacia *was isolated, are shown in Table 1. In four patients specimens were obtained in the ICU, whereas in the remaining patient the pathogen was isolated in a urine sample collected before ICU admission.

**Table 1:** Characteristics of patients and their evolution since hospital admission until *B cepacia *isolation and ICU discharge

Data	Study patients				
	
	1	2	3	4	5
Age (years)	78	75	78	85	71
Diagnosis	Peritonitis	Heat stroke	Heat stroke	Urinary septic shock	Peritonitis and cardiac arrest
Hospital admission	14 July 2006	28 July 2006	26 July 2006	3 August 2006	11 July 2006
ICU admission	15 July 2006	28 July 2006	26 July 2006	8 August 2006	21 August 2006
*B cepacia *isolation	1 August 2006	3 August 2006	12 August 2006	12 August 2006	18 August 2006
Sample 1	TA^a^	TA	CVC/skin	AC/blood	Urine
Sample 2	TA^a ^(5 August 2006)	TA^a ^(5 August 2006)	Urine (14 August 2006)	Urine (15 August 2006)	CVC/Blood, urine (12 September 2006)
Sample 3	TA^a ^(18 August 2006)	TA^a ^(16 August 2006)	-	-	-
Sample 4	TA^a ^(20 August 2006)	TA^a ^(21 August 2006)	-	-	-
Sample 5	TA^a^/CVC (19 August 2006)	Blood*/CVC (24 August 2006)	-	-	-
ICU discharge	9 September 2006	28 August 2006	18 September 2006	23 August 2006	17 September 2006
Cause of death	MOF	Encephalopathy	Alive	Alive	Encephalopathy
B. cepacia Related death	No	No			No

^a^Together with one or more micro-organisms. AC, arterial catheter; CVC, central venous catheter; ICU, intensive care unit; MOF, multiple organ failure; TA, tracheal aspirated.

Isolation of *B. cepacia *was associated with bacteraemia in three patients, lower respiratory tract infection in one and urinary tract infection in one. The cause of bacteraemia was attributed to a respiratory source in one case and to a central venous catheter in one; the remaining case was considered a primary bacteraemia. In three patients, new *B. cepacia *strains were isolated in control samples (on two occasions from the same tracheal aspirate samples as the original specimen, and in one patient, with initial positive samples from a central venous catheter and peripheral blood, *B. cepacia *was later isolated from urine samples). In the two patients with *B. cepacia *recovered from tracheal aspirate samples, the infective strain persisted despite directed antibiotic treatment. In one of these patients, *B. cepacia *along with *Pseudomonas aeruginosa *were isolated in blood cultures 2 weeks later, and in another patient from a central venous catheter tip and pharyngeal swab. Finally, another patient with initial urinary tract infection exhibited mixed bacteremia (*B. cepacia *and *P. aeruginosa*) in the final stage of the clinical course. Surveillance samples drawn from adjacent patients with an artificial airway were negative for the epidemic strain.

**Figure 1:**
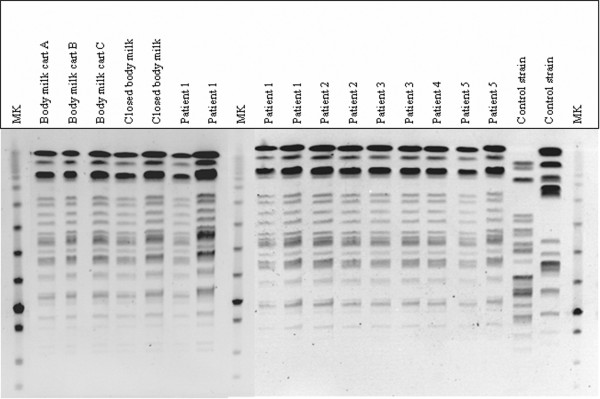
PFGE pattern of *Burkholderia cepacia *isolates in body milk and biological samples. MK, molecular weight marker.

In order to assess whether moisturizer had been contaminated before or after opening of the jar, three new hermetically closed units stored in the hospital pharmacy service or in the ICU, from three different batches (one of them coinciding with that analyzed in the ICU), were sent for culture. In samples obtained from two moisturizing body milk units – one belonging to the batch from which the initial isolation of the micro-organism has been obtained – *B. cepacia *strains were isolated (Table 2). Quantitative data regarding contamination of the moisturizing body milk were not obtained. Strains isolated from environmental and biological samples were identified as belonging to the same clone by PFGE (Figure 1).

**Table 2:** Results of cultures of ICU environmental samples (fluids)

Sample	Result
Phase I study (16 August 2006)	
Iodine solution (cart A: open)	Negative
Iodine solution (cart B: open)	Negative
Eau de Cologne (cart A: open)	Negative
Eau de Cologne (cart B: open)	Negative
Eau de Cologne (cart C: open)	Negative
Moisturizing body milk (cart A: open): batch 527.05–06	*Burkholderia cepacia*
Moisturizing body milk (cart B: open): batch 527.05–06	*Burkholderia cepacia*
Moisturizing body milk (cart C: open): batch 527.05–06	*Burkholderia cepacia*
Phase II study (25 August 2006)	
Moisturizing body milk (ICU: closed): batch 527.05.06	*Burkholderia cepacia*
Moisturizing body milk (pharmacy: closed): batch 512.07.06	*Burkholderia cepacia*
Moisturizing body milk (ICU: closed): batch 525.03–06	Negative

ICU, intensive care unit.

Once it was suspected that the moisturizer was the source of the outbreak the cream was withdrawn from the ICU (23 August 2006), and when samples from closed units were positive for the same pathogen by molecular typing (3 September 2006) the cream was withdrawn from all hospitalization units. Notification was immediately sent to the manufacturer and the Ministry of Health and Consumption. No new cases of *B. cepacia *infection occurred in the hospital.

## Discussion

The main contribution of the present study is the identification of a new reservoir of nosocomial pathogens, in this case *B. cepacia *in the moisturizing body milk used in the care of bedridden ICU patients. In this case, the epidemiological chain began with contamination of the lotion during manufacturing, transportation, or storing stages before application of moisturizing body milk to patients. Then, the hands of nursing personnel transmitted the pathogen to patients, in whom contamination of inert devices (catheters or tubes) or direct administration (skin, wounds, or airways) was responsible for severe nosocomial infection.

The implementation in our hospital of continuous control of pathogens of significance, among which nonfermenting Gram-negative bacilli are included, allowed us not only to detect the outbreak promptly but also to study environmental samples, facilitating the identification and elimination of the reservoir.

The standard procedure for study of an epidemic outbreak in our hospital is based on a case-control study. In the present case, however, samples of various products routinely used in the care of ICU patients were analyzed. Suspicion was based on the fact that infected patients did not occupy adjacent beds and that use of products found in treatment carts at the time of the outbreak was a characteristic common to all affected patients. In nonbiological samples, *B. cepacia *was isolated in three samples of the moisturizing body milk that was applied to the patients and available for use in ICU treatment carts. In other nonbiological samples sent for culture, no pathogens were isolated.

Topical products for skin care are not required to be sterilized [10]. The microbiological quality of these products is regulated by the European Pharmacopoeia, topical products (category II: nonsterile), which indicates that topical products should not contain more than 10^2 ^aerobic bacteria or moulds, and no more than 10^1 ^enterobacteria per gram or millilitre, as well as complete absence of *P. aeruginosa *and *Staphylococcus aureus*. In the outbreak reported here, no quantitative studies were performed but growth of forbidden species was not detected. B. cepacia is a nonfermenting Gram-negative bacillus equal to *P. aeruginosa*, so that presumably no strains of this pathogen would have been detected.

It has traditionally been suggested that the appearance of multiresistant pathogens is related to the use of broad-spectrum antibiotics over prolonged periods of time. Although in most cases this is the main mechanism of selection, in the cases reported here *B cepacia *was acquired from an exogenous source from an external reservoir introduced into the ICU. Intrinsic contamination of nasal sprays [5,11,12] and disinfectants [8] with *B. cepacia *has previously been documented, but the outbreak reported here is the first observation of *B. cepacia *infection secondary to contamination of a cosmetic product.

Accumulation of colonized and/or infected patients in the ICU despite distribution of the contaminated batch of the body milk throughout the hospital wards may be accounted for by two factors. First, maintenance of good body hygiene in ICU patients is carried out in the patient's own bed, and it is common practice to apply moisturizers after each manoeuvre that involves washing of cutaneous surfaces, so that a greater inoculum is obtained as compared with any other patients hospitalized in the wards. Second, because the number of samples from ICU patients submitted for culture – including surveillance samples – is much greater than for non-ICU patients, the probability of detection is also greater.

In most occasions in which products of common use are applied to patients (creams, antiseptic solutions, and so on), contamination and development of a reservoir results from handling of these products by health care personnel. In the present outbreak, however, contamination of the moisturizer occurred during the manufacturing process.

## Conclusion

An outbreak of *B. cepacia *infection in a multidisciplinary ICU was detected because of a well functioning hospital surveillance system for multiresistant pathogens. This outbreak of nosocomial infection caused by *B. cepacia *in five severely ill patients, in which moisturizing body milk was the reservoir of the causative pathogen, supports a strong recommendation not to use cosmetic products for which there is no guarantee of sterilization during the manufacturing process.

## Key messages

• Simultaneous identification of five ICU patients with infections caused by *B. cepacia *suggested the occurrence of an epidemic outbreak; a surveillance system for identification of multiresistant pathogens facilitated recognition of cases.

• The study of environmental samples allowed identification of the moisturizing body milk used in the patients' care as the reservoir of *B. cepacia*.

• All strains recovered from environmental and biological samples were identified as belonging to the same clone by PFGE.

• Contamination of the moisturizer occurred during the manufacturing process.

• Products used in the daily hygiene of critically ill patients should be sterile.

## Abbreviations

ICU = intensive care unit; PFGE = ulsed field gel electrophoresis.

## Competing interests

The authors declare that they have no competing interests.

## Authors' contributions

FAL designed the study, was involved in the care of patients, reviewed the literature, coordinated the study and drafted the manuscript. EM and TR were involved in the care of the patients. CS performed identification of the causative pathogen (genus and species) in clinical samples. IP performed identification of the causative pathogen (genus and species) of environmental samples. PC performed the molecular studies. HK was involved in the programme of surveillance and control of multiresistant pathogens in the hospital. AV was involved in the care of the patients and made contributions to the initial drafts. All authors read and approved the final manuscript.
